# Proteomics informed by transcriptomics reveals Hendra virus sensitizes bat cells to TRAIL-mediated apoptosis

**DOI:** 10.1186/s13059-014-0532-x

**Published:** 2014-11-15

**Authors:** James W Wynne, Brian J Shiell, Glenn A Marsh, Victoria Boyd, Jennifer A Harper, Kate Heesom, Paul Monaghan, Peng Zhou, Jean Payne, Reuben Klein, Shawn Todd, Lawrence Mok, Diane Green, John Bingham, Mary Tachedjian, Michelle L Baker, David Matthews, Lin-Fa Wang

**Affiliations:** CSIRO Biosecurity Flagship, Australian Animal Health Laboratory, East Geelong, VIC Australia; Department of Cellular and Molecular Medicine, School of Medical Sciences, University of Bristol, Bristol, UK; Program in Emerging Infectious Diseases, Duke–National University of Singapore Graduate Medical School, Singapore, Singapore

## Abstract

**Background:**

Bats are a major reservoir of emerging infectious viruses. Many of these viruses are highly pathogenic to humans however bats remain asymptomatic. The mechanism by which bats control viral replication is unknown. Here we utilize an integrated approach of proteomics informed by transcriptomics to compare the response of immortalized bat and human cells following infection with the highly pathogenic bat-borne Hendra virus (HeV).

**Results:**

The host response between the cell lines was significantly different at both the mRNA and protein levels. Human cells demonstrated minimal response eight hours post infection, followed by a global suppression of mRNA and protein abundance. Bat cells demonstrated a robust immune response eight hours post infection, which led to the up-regulation of apoptosis pathways, mediated through the tumor necrosis factor-related apoptosis inducing ligand (TRAIL). HeV sensitized bat cells to TRAIL-mediated apoptosis, by up-regulating death receptor transcripts. At 48 and 72 hours post infection, bat cells demonstrated a significant increase in apoptotic cell death.

**Conclusions:**

This is the first study to comprehensively compare the response of bat and human cells to a highly pathogenic zoonotic virus. An early induction of innate immune processes followed by apoptosis of virally infected bat cells highlights the possible involvement of programmed cell death in the host response. Our study shows for the first time a side-by-side high-throughput analysis of a dangerous zoonotic virus in cell lines derived from humans and the natural bat host. This enables a way to search for divergent mechanisms at a molecular level that may influence host pathogenesis.

**Electronic supplementary material:**

The online version of this article (doi:10.1186/s13059-014-0532-x) contains supplementary material, which is available to authorized users.

## Background

Emerging infectious diseases pose a significant threat to human and animal welfare. Many emerging and re-emerging infectious diseases are zoonoses derived from wildlife, particularly bats [1,2]. Bats are now recognized as a major reservoir of zoonotic agents. High profile examples include the henipaviruses (Hendra and Nipah) [3-5], severe acute respiratory syndrome-like coronavirus [6,7], Ebola virus [8] and most recently the Middle East respiratory syndrome coronavirus [9,10]. The significance of bats as a reservoir for zoonotic viruses was first recognized with the emergence of Hendra virus (HeV) in northern Australia in 1994. In two independent spillover events, HeV claimed the lives of 15 horses and two humans [3,4]. Approximately four years after HeV emerged, a related paramyxovirus, designated Nipah virus (NiV), emerged in farmed pigs in Malaysia. Between 1998 and 1999, this virus claimed the lives of 105 humans and resulted in the culling of over one million pigs [5]. NiV outbreaks occur annually in Bangladesh with cases of direct human-to-human transmission also reported. Bats of the *Pteropus* genus are the natural reservoir of both HeV and NiV.

Despite the fact that many of the zoonotic viruses harbored by bats are highly pathogenic to their spillover hosts, bats remain clinically unaffected and rarely display any signs of disease. Some rabies-like viruses are the notable exception [11,12]. The mechanism by which bats control viral replication remains largely unknown. Despite the absence of clinical disease, bats are capable of shedding virus and triggering subsequent zoonotic transmission. This situation implies bats are capable of controlling viral replication, but not eliminating it. Studies on Ebola have demonstrated that bat lung fibroblasts (derived from the Mexican free-tailed bat) are capable of maintaining a low-level persistent infection with wild-type Ebola Zaire [13]. Recent studies have demonstrated that genes involved in innate immunity have evolved rapidly under positive selection within the Australian black flying fox (*Pteropus alecto*), indicative of co-evolution between virus and host [14]. However, in-depth knowledge about the antiviral response of bats is still lacking, as are the reagents necessary to study these non-model species. The present study addresses this knowledge gap by comparing the response of *P. alecto* with humans following HeV infection. As the natural reservoir of HeV, *P. alecto* remains clinically asymptomatic. By contrast, zoonotic transmission of HeV to horses and humans is often fatal [15].

Genomic resources are now available for a number of bat species, including whole draft genome sequences [14,16-18] and *de novo* assembled transcriptomes [19,20]. A draft genome sequence for the *P. alecto* was released in 2013 [14]. However, to date, no studies have examined the antiviral response of this species - or any other bat species - to infectious viruses at either the transcriptome or proteome level. The study of infectious agents in any non-model organism by high-throughput techniques is severely constrained by the quality and availability of gene model annotations, particularly in the field of proteomics. While the draft *P. alecto* genome was annotated using a combination of homology, *de novo* prediction and transcriptomics [14], continual refinement is necessary. To circumvent the reliance on high-quality annotation models, we recently developed proteomics informed by transcriptomics (PIT) analysis. This technique collects RNA-sequencing (RNAseq) and quantitative high-throughput proteomics data simultaneously, then uses the transcriptomic data to refine and inform the proteomics analysis. We have previously demonstrated that this combined approach sidesteps the issue of bioinformatics annotation and enables the analysis of any species on a similar footing to humans [21].

Using stable isotope incorporation of amino acids in cell culture (SILAC) and RNAseq transcriptomics, we compared the response of kidney cells derived from human and the Australian black flying fox to HeV infection at 8 and 24 h. Because of the paucity of well-characterized primary cell lines from the Australian black flying fox, we chose to utilize immortalized cells lines from bats (known as PaKiT03) and humans (HEK293T). Attempts were made to choose cells that were transcriptionally and phenotypically similar. Both cell lines were derived from the kidney, immortalized using viral antigens, and are supportive of HeV replication.

We found that the response of HEK293T cells was significantly different to PaKiT03 cells. The HEK293T cells showed little transcriptional or proteomic response to HeV at 8 h post infection (hpi), followed by a global suppression in protein abundance at 24 hpi. By contrast, PaKiT03 cells initially underwent activation of NF-kappa-B (NF-κB) at 8 hpi, followed by the induction of extrinsic apoptosis pathways at 24 hpi. Further analysis of the responses after 48 and 72 h of HeV infection revealed that PaKiT03 cells, but not HEK293T cells, underwent apoptotic cell death. In addition, our study provides proteomic evidence for over 5,000 genes in the *P. alecto* genome, including several hundred previously unannotated genes. To our knowledge, this is the first study to examine the transcriptional and proteomic response to HeV in any cell type. Furthermore, this study represents the first side-by-side transcriptomic and proteomic analysis of a single virus in two relevant host species. This study generates valuable knowledge concerning the divergent response of a reservoir and susceptible host to a highly pathogenic bat-borne virus.

## Results

### HeV transcription comparison between cell lines

**Figure 1:**
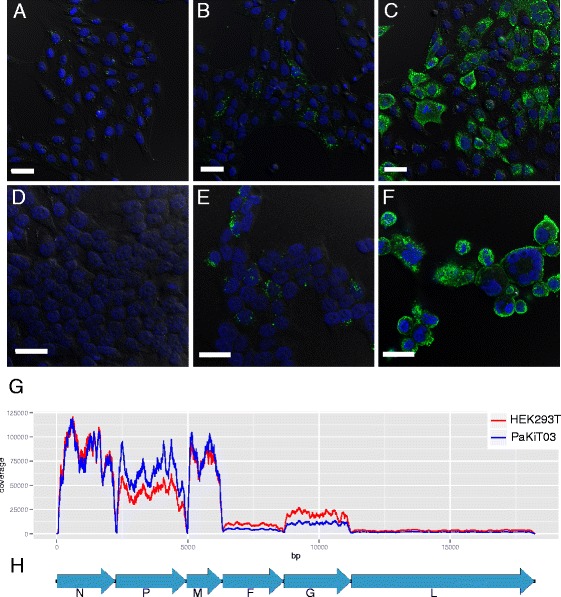
**HeV infection and transcription in bat and human cells.** Confocal microscopy was used to visualize HeV-infected cells. Nuclei are stained with 4′,6-diamidino-2-phenylindole dihydrochloride, and HeV-N protein was immunodetected with an anti-N polyclonal antibody. PaKiT03 cells at **(A)** 0 hpi, **(B)** 8 hpi and **(C)** 24 hpi are shown. HEK293T cells infected with HeV for **(D)** 0 hpi, **(E)** 8 hpi and **(F)** 24 hpi are shown. Scale bar is 30 μm. **(G)** Transcription profile of HeV in PaKiT03 and HEK293T at 24 hpi. **(H)** Genome structure of HeV. bp, base pairs; F, fusion protein; G, glycoprotein (attachment protein); L, large protein (polymerase); M, matrix protein; N, nucleocapsid; P, phosphoroprotein (includes proteins V, W and C).

Virus nucleocapsid (N) protein was detectable in increasing numbers of cells at 8 and 24 hpi (Figure 1A-F). Viral-induced syncytia were observed at 24 hpi in HEK293T cells (Figure 1F), but not in PaKiT03 cells (Figure 1C). RNAseq reads from both cell lines were mapped to the HeV genome using TopHat. We randomly selected one million mapped reads from PaKiT03 and HEK293T cells at 24 hpi to enable a side-by-side comparison of levels of expression from HeV genes in each cell line (Figure 1G). Comparing gene expression in the two species cell lines revealed similar numbers of reads mapped against the N and matrix protein (M), with differences in all the other genes (Figure 1G,H). A steep decline in the abundance of transcripts at the M-F gene boundary was observed in both cell lines.

### HeV induces differential gene and protein expression

**Figure 2:**
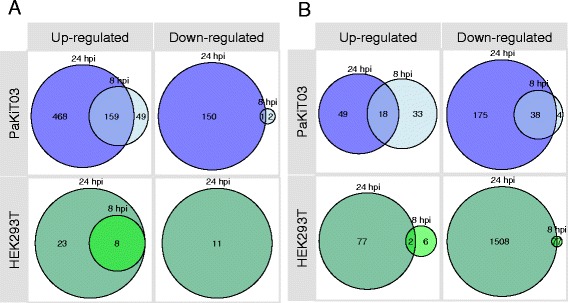
**Statistics of differential expression. (A)** Differential expression of Trinity transcripts at 8 and 24 hpi was assessed as pairwise comparisons to 0 hpi. The number and overlap of significantly differentially expressed transcripts (adjusted *P* <0.05) for 8 and 24 hpi are illustrated as two-way proportional Venn diagrams. Venn diagrams are organized in a 2 × 2 matrix where up- and down-regulated statistics are given in the columns and the cell type is given in the rows. **(B)** Differential expression of Trinity proteins were defined as proteinGroups with SILAC ratios ≥2 fold relative to 0 hpi. Two-way proportional Venn diagrams for proteins differentially expressed at 8 and/or 24 hpi are illustrated as for **(A)**.

A total of 222,569 and 285,918 transcripts (with a predicted open reading frame (ORF) >200 nucleotides) were assembled *de novo* for the PaKiT03 and HEK293T cells, respectively. Differential expression analysis of *de novo* assembled transcripts revealed that the overall response to HeV was distinct between cell lines (Figure 2A). In the PaKiT03 cells, HeV infection induced the expression of over 200 transcripts at 8 hpi and over 600 transcripts at 24 hpi (Figure 2A, Additional file 1). By contrast, only 8 and 31 transcripts were up-regulated at 8 and 24 hpi in the HEK293T cells (Figure 2A, Additional file 1). In both cell lines, many of the transcripts induced at 8 hpi remained up-regulated at 24 hpi. The proteomic response also differed significantly between the two cell lines (Figure 2B). Most notably, in HEK293T cells over 1,500 proteins were down-regulated at 24 hpi, whereas only 213 proteins were down-regulated in PaKiT03 cells at this time point (Additional file 1). Of the 100 proteins that were significantly induced in PaKiT03 cells at 8 and/or 24 hpi, 17 were also significantly up-regulated at the mRNA level (Additional file 1). Many of these proteins have proven roles in innate immunity, including the transcription factor CCAAT/enhancer-binding protein delta (CEBPD), the interferon (IFN)-stimulated gene ISG20, receptors CD40 and CD44, and complement component C3. By contrast, no proteins were up-regulated at both the protein and mRNA level in the HEK293T cells. Furthermore, with one exception in the PaKiT03 cells (SLC12A2), the proteins down-regulated at 8 and/or 24 hpi were not significantly down-regulated at the mRNA level for either cell line. A full list of Trinity transcript and protein expression statistics are provided as Additional files 2 (PaKiT03) and 3 (HEK293T).

Using our PIT-based approach we were able to detect a large number of genes and proteins not currently in the UniProt lists for *P. alecto*. A total of 263 Trinity-specific proteinGroups were identified, which represent potentially novel or alternative protein-coding genes previously not identified in the *P. alecto* genome. Two notable genes identified by this strategy, that were also differentially expressed, were *CEBPD* and the gene for tumor necrosis factor (TNF) receptor superfamily 26 (*TNFRSF26*).

### Divergent responses of orthologous transcripts

**Figure 3:**
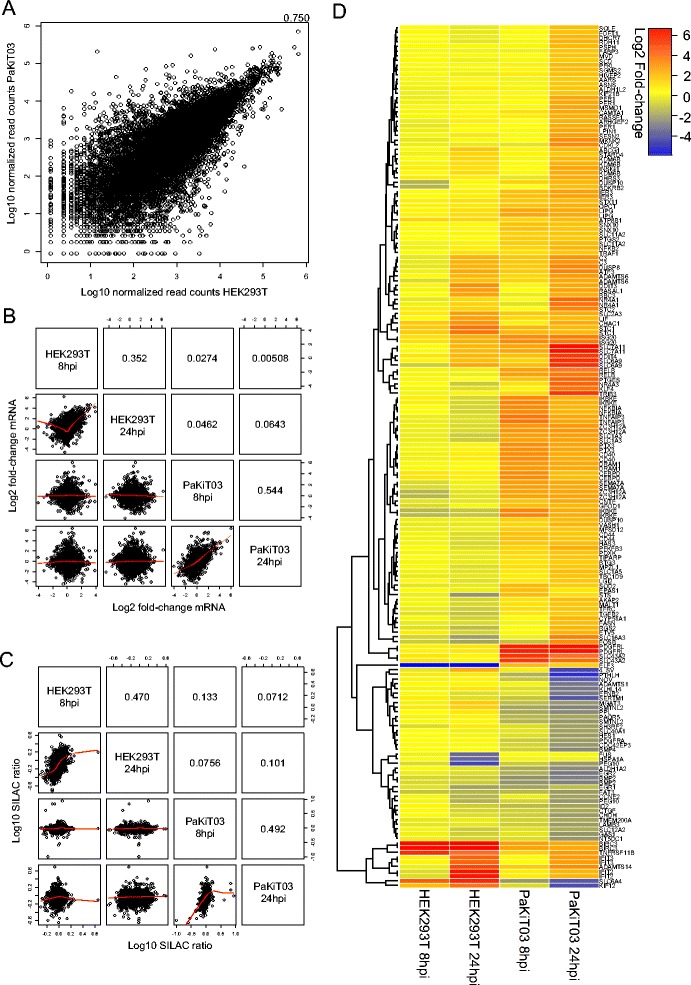
**Relationship between mRNA and protein expression of orthologous transcripts. (A)** Scatterplot illustrating the relationship in baseline (0 hpi) mRNA expression as log10 normalized fragments per kilobase of transcript per million mapped reads (FPKM) for all ortholog transcripts (identity >70%). Pearson’s correlation coefficient is indicated in the top right hand corner. **(B)** Scatterplot matrix of the correlation between transcript expression (log2-fold change) at 8 and 24 hpi in PaKiT03 and HEK293T cells. Pearson’s correlation coefficients are given in upper boxes. Simple linear regression models were fitted and are illustrated as red lines through each scatterplot. **(C)** Scatterplot matrix of the correlation between protein expression (log10 SILAC ratio) at 8 and 24 hpi in PaKiT03 and HEK293T cells. Pearson’s correlation coefficients are given in the upper boxes. Simple linear regression models were fitted and are illustrated as red lines through each scatterplot. **(D)** Heatmap of orthologous transcripts that were significantly differentially expressed at 8 and/or 24 hpi in either cell line. Expression values for transcripts are given as log2-fold change.

Next we identified and compared the expression of orthologous transcripts between the PaKiT03 and HEK293T cells. We used the RNAseq data to *de novo* derive transcripts for both cell lines. All PaKiT03 and HEK293T Trinity transcripts with >70% similarities to the same human gene were obtained by Basic Local Alignment Search Tool (BLAST). Over 10,000 orthologous transcripts were identified by this strategy (Additional file 4). We first examined the correlation in baseline mRNA expression (at 0 hpi) between the cell lines (Figure 3A). Because SILAC is a relative quantification method we could not compare the baseline protein expression between the cell lines. A Pearson’s correlation coefficient of 0.75 demonstrated that orthologous transcripts showed similar baseline mRNA expression prior to HeV infection. Despite this, at 8 and 24 hpi the correlation in mRNA expression between orthologous transcripts fell significantly (Figure 3B). Similarly, at the protein level there was poor correlation in relative protein abundance of orthologous transcripts at 8 and 24 hpi (Figure 3C). Moderate agreement in mRNA fold change and relative protein abundance was, however, observed between 8 and 24 hpi within each cell line (Figure 3B,C). A heatmap of all orthologous transcripts that were significantly differentially expressed - at one or more time point – confirmed that the response was similar at 8 and 24 hpi within each cell line, but differed between cell lines (Figure 3D).

### Enrichment of immune and TNF apoptotic pathways in PaKiT03 cells

**Figure 4:**
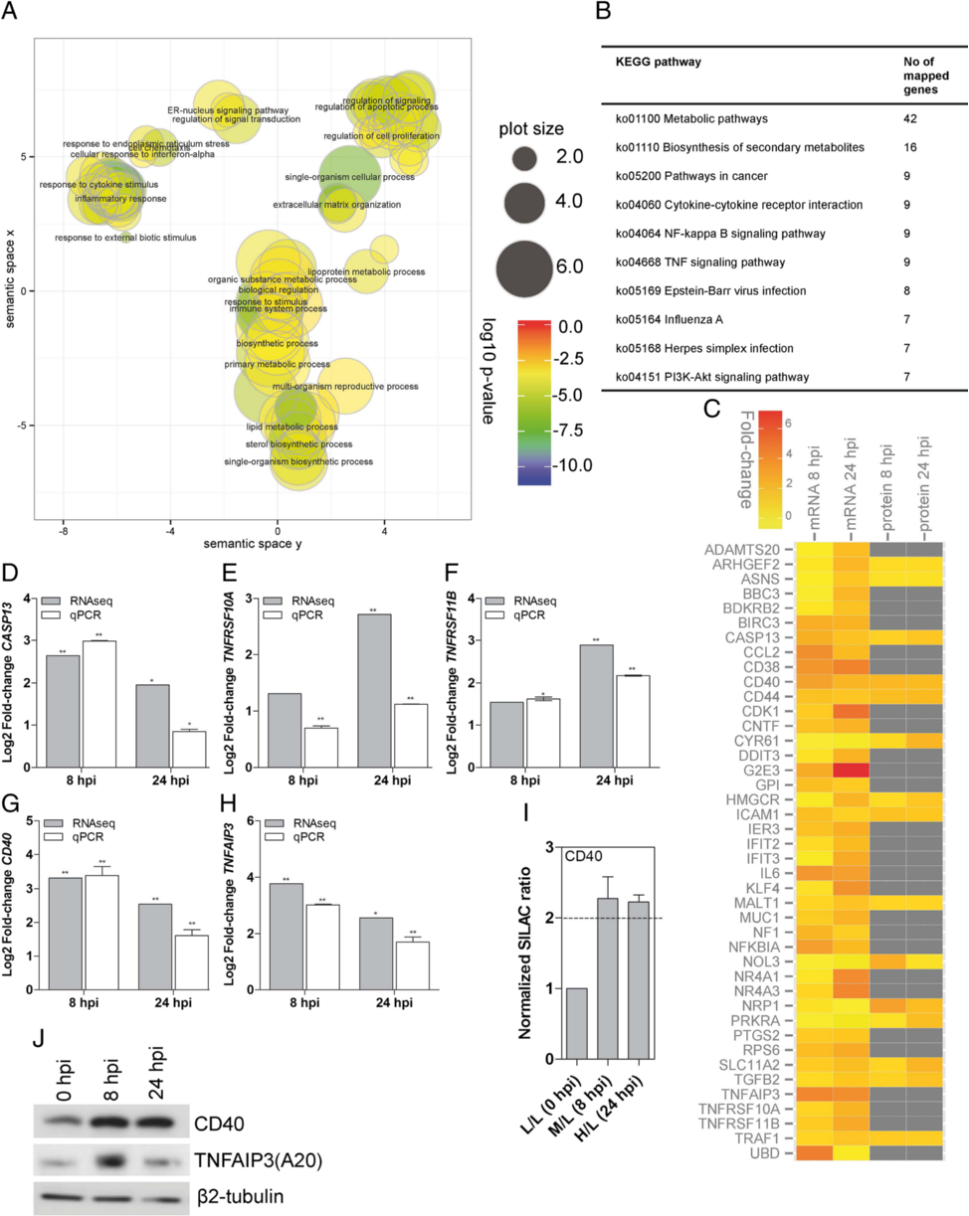
**Activation of apoptosis pathways in HeV-infected PaKiT03 cells. (A)** GO terms enriched in the up-regulated genes/proteins in PaKiT03 cells were analyzed and visualized using REVIGO, where redundant GO terms are removed. The size of the circle represents the number of enriched child GO terms contributing to the parent term. The circle color represents the uncorrected log10 *P*-value for each parent GO term. Semantic space is the result of multi-dimensional scaling where similar GO terms cluster together. A full list of enriched GO terms, including false discover rate-corrected *P*-values, are presented in Additional file 5. **(B)** Top 10 most over-represented KEGG pathways, and the number of significantly differentially expressed genes/proteins contributing to each pathway. The full list of over-represented KEGG pathways is presented in Additional file 6. **(C)** Expression profile of significantly up-regulated genes/proteins in the PaKiT03 cells which were assigned to the GO term regulation of apoptotic process [GO:0042981] or apoptotic process [GO:0006915]. Gray cells represent proteins where no peptides were observed. Expression values for mRNA are given as log2-fold change, while expression values for proteins are normalized SILAC ratios. Validation of RNAseq differential expression with real-time quantitative PCR (qPCR) for **(D)** *CASP13*, **(E)** *TNFRSF10A*, **(F)** *TNFRSF11B*, **(G)** *CD40* and **(H)** *TNFAIP3/A20* in PaKiT03 cells. Real-time PCR differential expression was calculated based on relative expression to 0 hpi and normalized to *GAPDH*. Log2-fold change is presented; *P <0.05, **P <0.01. **(I)** Relative protein expression profile of CD40 in PaKiT03 cells measured by SILAC. **(J)** Protein expression profile of CD40 and TNFAIP3/A20 in PaKiT03 cells measured by Western blot. β2-tubulin served as the load control.

Gene Ontology (GO) enrichment analysis was performed on the combined list of transcripts and proteins that were either up- or down-regulated at one or more time points. Proteins and transcripts that were up-regulated in the PaKiT03 cells at 8 and/or 24 hpi were broadly involved in response to external stimulus [GO:0009605], response to stress [GO:0006950], defense response [GO:0006952], regulation of apoptotic processes [GO:0042981] and immune system process [GO:0002376] (Figure 4A, Additional file 5). Transcript and protein sets were also subjected to Kyoto Encyclopedia of Genes and Genomes (KEGG) pathway mapping. Many of the up-regulated PaKiT03 transcripts and proteins were mapped to TNF signaling (ko4668), NF-κB signaling (ko04064) and cytokine-cytokine receptor interaction (ko04060) pathways (Figure 4B, Additional file 6).

A subset of transcripts involved in apoptosis and TNF signaling (Figure 4C), including *TNFRSF10A* (*DR4*), *TNFRSF11B*, *CD40* (also known as *TNFR5*), *CASP13* and *TNFAIP3* (*A20*), were chosen for validation using quantitative PCR (qPCR). In all cases, the differential expression was confirmed, although the magnitude of the fold change was less by qPCR compared to RNAseq (Figure 4D-H). Due to the paucity of bat specific and cross-reactive antibodies, we could only examine the expression of two proteins - CD40 and TNFAIP3 - by Western blotting in the PaKiT03 cells. CD40 was up-regulated over two-fold at the protein level in the SILAC analysis (Figure 4I). No peptides were found for TNFAIP3 within the SILAC analysis, however considering its mRNA was significantly up-regulated, we examined its protein expression with Western blot nonetheless. Both proteins demonstrated differential expression at either 8 and/or 24 hpi, similar to their mRNA expression (Figure 4J).

### Interferon production and signaling in HEK293T cells

**Figure 5:**
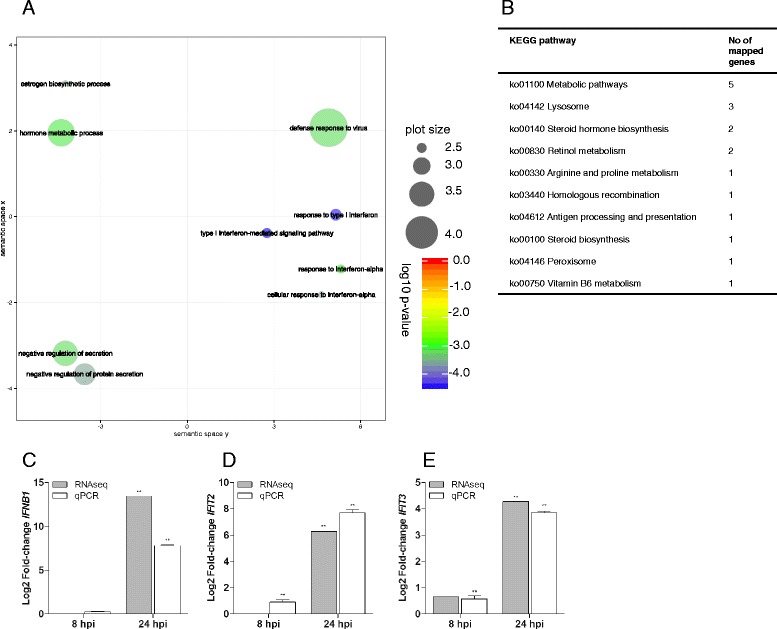
**Interferon production and signaling in HeV-infected HEK293T cells. (A)** GO terms enriched in the up-regulated genes/proteins in HEK293T cells were analyzed and visualized using REVIGO, where redundant GO terms are removed. The size of the circle represents the number of enriched child GO terms contributing to the parent term. The circle color represents the uncorrected log10 *P*-value for each parent GO term. Semantic space is the result of multi-dimensional scaling where similar GO terms cluster together. A full list of enriched GO terms, including false discover rate-corrected *P*-values are presented in Additional file 5. **(B)** Top 10 most over-represented KEGG pathways, and the number of significantly differentially expressed genes/proteins contributing to each pathway. The full list of over-represented KEGG pathways is presented in Additional file 6. Validation of RNAseq differential expression with real-time qPCR for **(C)** *IFNB1*, **(D)** *IFIT2* and **(E)** *IFIT3*. Real-time PCR differential expression was calculated based on relative expression to 0 hpi and normalized to *GAPDH*. Log2-fold change is presented; *P <0.05, **P <0.01.

GO enrichment of HEK293T proteins and transcripts up-regulated at 8 and/or 24 hpi revealed enrichment for only a few biological processes, including response to type I IFN [GO:0034340] and defense response to virus [GO:0051607] (Figure 5A, Additional file 5). Few transcripts and proteins from the HEK293T up-regulated set were mapped to known KEGG pathways, which included the lysosome pathway (ko04142) (Figure 5B, Additional file 6). We validated the expression of three human transcripts (*IFNB1, IFIT2* and *IFIT3*) that contributed to the over-representation of the type I IFN response. All three transcripts demonstrated significant up-regulation at 24 hpi by qPCR (Figure 5C-E).

### HeV up-regulates pro-apoptotic pathways in PaKiT03 cells

**Figure 6:**
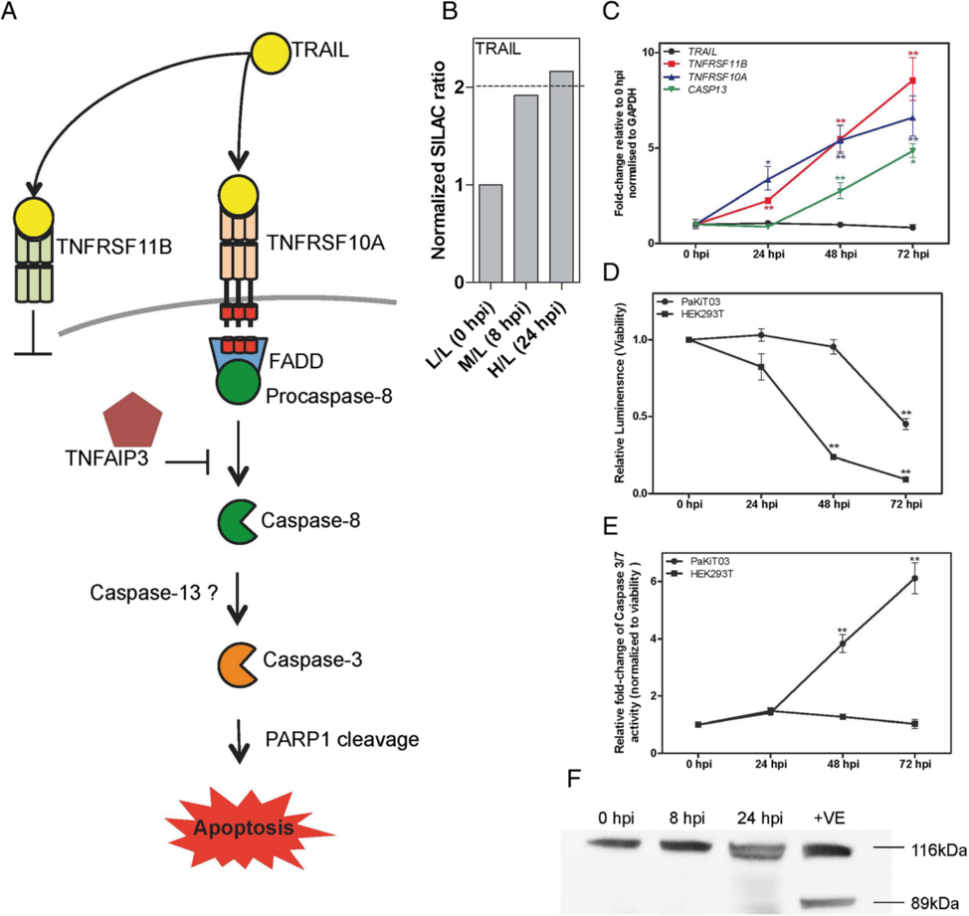
**Hendra virus induces apoptosis in PaKiT03, but not HEK293T cells. (A)** TRAIL-mediated apoptosis pathway. **(B)** TRAIL relative protein expression in PaKiT03 cells measured by SILAC. **(C)** mRNA fold change relative to 0 hpi and normalized to *GAPDH* of *TRAIL*, *TNFRSF10A*, *TNFRSF11B* and *CASP13* in PaKiT03 cells at 24, 48 and 72 hpi. **(D)** Cell viability relative to 0 hpi of PaKiT03 and HEK293T at 24, 48 and 72 hpi with HeV at a multiplicity of infection (MOI) of 5. One-way analysis of variance (ANOVA) was used to compare cell viability between time points. **(E)** Relative activity of caspase 3/7 normalized to cell viability for PaKiT03 and HEK293T at 0, 24, 48 and 72 hpi with HeV at an MOI of 5. One-way ANOVA was used compared the relative activity of caspase 3/7 between the PaKiT03 and HEK293T cells at each time point. **(F)** Western blot of PARP1 cleavage at 0, 8 and 24 hpi in HEK293T. The human PARP1 antibody was not cross-reactive with the PaKiT03 cells. *P <0.05, **P <0.01.

Considering the enrichment of apoptosis-related genes within the PaKiT03 cells, we next examined if the expression of transcripts and proteins involved in the apoptotic response differed between the cell lines. Apoptosis, or programmed cell death, is an important mechanism by which the host can eliminate virus-infected cells. Apoptosis can be triggered through intrinsic (mitochondrial-dependent) or extrinsic signaling pathways. In the present study, a number of genes/proteins involved in the extrinsic TNF receptor-associated inducing ligand (TRAIL)-mediated apoptosis signaling pathway were induced at 24 hpi in the PaKiT03 but not HEK293T cells (Figure 4C). TRAIL-mediated apoptosis occurs through binding of the trimerized ligand to its functional death receptor (DR4/TNFRSF10A or DR5/TNFRSF10B). This is followed by recruitment of the Fas-associated death domain, mediated through a death domain, which induces the sequential activation of caspase 8 (from its pro-caspase precursor) and caspase 3, ultimately leading to apoptosis-mediated cell death (Figure 6A)

.

A single peptide derived from the *P. alecto* TRAIL protein was found to be induced over 2-fold at 24 hpi in the PaKiT03 cells (Figure 6B). The fact that TRAIL is often secreted as a soluble form could explain why we only observed a single peptide. This peptide was detected when spectra were searched against the *P. alecto* official UniProt list of proteins and not the Trinity transcriptome-derived protein list. Furthermore, mRNA up-regulation of the functional TRAIL receptor (*TNFRSF10A/DR4*) was demonstrated by RNAseq and qPCR at 8 and 24 hpi (Figure 4E). In a longer time course experiment we demonstrated that *TNFRSF10A/DR4* mRNA, but not *TRAIL*, was further induced at 48 and 72 hpi (Figure 6C). A transcript encoding the pro-apoptotic enzyme caspase 13 was also induced at 8 and 24 hpi in the PaKiT03 cells (Figure 4D). Caspase 13 mRNA was also further induced at 48 and 72 hpi in the PaKiT03 cells (Figure 6C). Other genes and proteins with proven roles in intrinsic apoptosis processes, such as bcl2 binding component 3 (*BBC3*) and baculoviral IAP repeat containing 3 (*BIRC3*), were also induced in PaKiT03 cells in response to HeV (Figure 4C).

### Inhibitors of apoptosis in PaKiT03 cells

Interestingly, we also observed up-regulation of the two anti-apoptotic components within HeV-infected PaKiT03 cells, the TRAIL decoy receptor osteoprotegerin (*TNFRSF11B*) and the ubiquitin ligase A20 (*TNFIAP3*). *TNFRSF11B* was induced at the mRNA level at both 8 and 24 hpi (Figure 4F). To further examine the role of anti-apoptotic components, a long time course experiment was performed on PaKiT03 cells. *TNFRSF11B* continued to be up-regulated at 48 and 72 hpi (Figure 6C). Acting as a decoy receptor, TNFRSF11B is capable of inhibiting TRAIL-mediated apoptosis as well as NF-κB activation. In a similar vein, TNFIAP3 also inhibits TRAIL-induced apoptosis in certain cells, through poly ubiquitination of the receptor-interacting protein 1 (RIP1) and inhibition of caspase 8 cleavage. In this study *TNFIAP3* mRNA was significantly induced at 8 and, to a lesser extent, 24 hpi in PaKiT03 cells, but not in HEK293T cells (Figure 4H). Western blot of TNFIAP3 revealed an increase in protein abundance at 8 hpi in bat cells (Figure 4J). TNFIAP3 itself is an NF-κB-activated protein that plays an important role in suppressing NF-κB activity through a negative feedback mechanism. Again, no differential expression of *TNFIAP3* mRNA/protein was observed in the HEK293T cells.

### Anti-apoptotic signals in HEK293T cells

In contrast to the up-regulation of TRAIL and its down-stream components in PaKiT03 cells, a down-regulation of proteins involved in both the intrinsic and extrinsic apoptosis pathways was observed in HEK293T cells at 24 hpi. Indeed, the pro-apoptotic proteins Bcl2 antagonist of cell death (BAD), caspase 2 and TNFR1-associated death domain protein were down-regulated at 24 hpi in HEK293T cells (Additional file 1). Interestingly, in the HEK293T cells the *TNFRSF11B* ortholog was also induced at 24 hpi (although not statistically significantly) (Figure 3D). However, the expression of *TNFRSF10A*/*DR4* and other functional TRAIL related genes/proteins remained stable in HeV-infected HEK293T cells (data not shown).

### HeV induces apoptotic cell death in PaKiT03 but not HEK293T cells

Considering many apoptotic components (mRNA and proteins) were up-regulated in PaKiT03 but not HEK293T cells, we next set out to test whether HeV actually induces functional apoptosis in either cell line. Apoptosis was assessed by measuring the activity of the caspase 3/7, the cleavage of PARP1 (HEK293T only) and terminal deoxynucleotidyl transferase dUTP nick end labeling (TUNEL). Both cell lines were infected at a multiplicity of infection (MOI) of 5 for 24, 48 and 72 h and apoptosis was measured by a Caspase-Glo®3/7 luminescence assay. Cytopathic effects typical of HeV were observed after 24 hpi and increased at 72 hpi (data not shown). To account for the decrease in cell viability within infected cells, we first measured cell viability using a CellTiter-Glo® luminescence assay and then normalized the caspase 3/7 luminescence to cell viability. As expected, cell viability decreased rapidly after 24 hpi in the HEK293T cells, but not in bat cells. At 24 hpi, only a small and non-significant decrease (*P* >0.05) in the viability of HEK293T cells was observed. At 48 hpi a significant decrease (*P* <0.01) in cell viability was observed in the HEK293T cells, which further decreased at 72 hpi (*P* <0.01). At 72 hpi the PaKiT03 cells decreased in viability by approximately 50% (Figure 6D).

**Figure 7:**
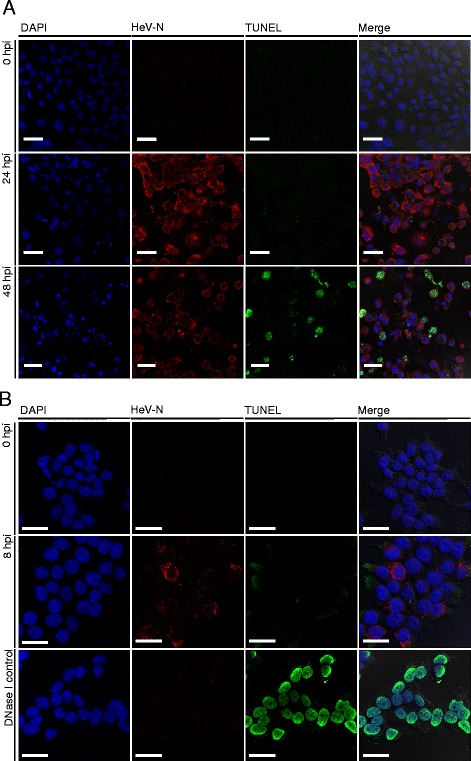
**TUNEL staining of HeV-infected (A) PaKiT03 and (B) HEK293T cells.** Cells were infected with HeV for either 8, 24 or 48 h using an MOI of 5. TUNEL staining was achieved using the Click-iT® TUNEL Alexa Fluor® 488 kit (green, fluorescence) and HeV-N was immunodetected as described above (red, fluorescence). DNase I treatment was used as a positive control on HEK293T cells that demonstrated clear nuclear staining. Owing to the decrease in cell viability at 24 hpi and beyond in the HEK293T cells, reliable TUNEL staining could not be performed after 8 hpi. Scale bar is 30 μm in all panels.

A significant increase in caspase 3/7 was observed at 48 and 72 hpi in the PaKiT03 compared to uninfected cells (*P* <0.01; Figure 6E). By contrast, no increase in caspase 3/7 was observed in HEK293T cells at any time point. Furthermore, we observed no cleavage of PARP1 in HEK293T cells at 8 or 24 hpi (Figure 6F). The observation that HeV significantly reduces cell viability of HEK293T cells after 24 hpi meant it was not possible to examine the apoptotic response after this time using either PARP1 Western blotting or TUNEL. TUNEL staining of HeV-infected PaKiT03 cells at 24 and 48 hpi demonstrated an increased number of apoptotic cells at 48 hpi (Figure 7A). This is in agreement with results from the caspase 3/7 assay. In most cases, TUNEL-positive cells were also infected with HeV at 48 hpi. TUNEL staining of HeV-infected HEK293T could only be performed at 0 and 8 hpi. After this time point HeV infection caused many cells to detach from the coverslips. TUNEL staining at 0 and 8 hpi revealed no apoptotic HEK293T cells (Figure 7B). This finding is consistent with the lack of PARP1 cleavage at 8 and 24 hpi in HEK293T cells. Weak cytoplasmic staining was observed in some HEK293T cells, but given we observed no nuclear staining (as seen in PaKiT03 and the DNase control) we concluded this was the result of non-specific background staining. The human PARP1 antibody was not cross-reactive in the bat cells, and so this method could not be used.

### HeV-induced apoptosis in additional human and bat cells

**Figure 8:**
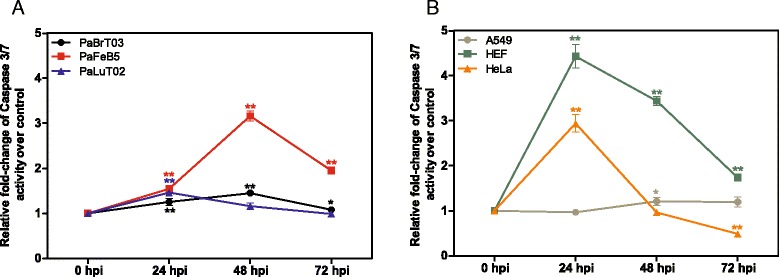
**Apoptosis in other (A) bat and (B) human cells.** Relative fold change of caspase 3/7 activity of bat cells from brain (PaBrT03), fetus (PaFeb5) and lung (PaLuT02), and common human cells from lung (A549), embryonic fibroblasts (HEF) and HeLa cells. All cells were infected with HeV for 24, 48 and 72 hpi at an MOI of 5. Uninfected cells served as the control for each time point. Two sample *t*-tests were used to compare the relative activity of caspase 3/7 between the control and infected cells at each time point. *P <0.05, **P <0.01.

While a clear difference in HeV-induced apoptosis was observed between the HEK293T and PaKiT03 cells, we concede that these phenotypes may not be representative of all human and bat cells. Given this limitation, we examined whether HeV induces apoptotic cell death across other human and bat cell types. Owing to the poorly characterized nature and paucity of primary bat cell lines, we concentrated on well-characterized immortalized cell lines from *P. alecto*. Apoptosis was assessed by the Caspase-Glo®3/7 luminescence assay. Following HeV infection we observed significant increased caspase 3/7 activities in *P. alecto* fetus cells (PaFeB5) and, to a lesser degree, brain cells (PaBrT03). Activities in both of these cell types were highest at 48 hpi (*P* <0.01, Figure 8A). In contrast to our previous observations in HEK293T cells, the human embryonic fibroblasts (HEF) and HeLa cells demonstrated a strong increase in caspase 3/7 activities at 24 hpi (*P* <0.01; Figure 8B). This response diminished in both HEF and HeLa cells at 48 and 72 hpi. A549 cells demonstrated only a slight but statistically significant increase in caspase 3/7 activity at 48 hpi (p <0.05; Figure 8B).

### HeV sensitizes PaKiT03 cells to TRAIL-mediated apoptosis

**Figure 9:**
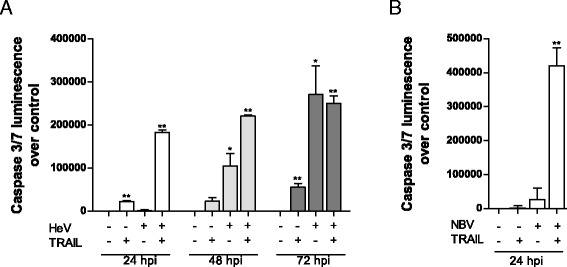
**HeV sensitizes PaKiT03 cells to TRAIL-mediated apoptosis.** Cells were either treated with 500 ng/ml human recombinant TRAIL and/or infected with virus (MOI = 5) for 24, 48 and 72 hpi. The activities of caspase 3/7 within the experimental samples were compared to the control (no TRAIL or virus) using one-way analysis of variance for each time point. *P <0.05, **P <0.01. All assays were performed in triplicate. **(A)** Increase of caspase 3/7 activity over control in PaKiT03 cells following stimulation with human recombinant TRAIL and/or HeV. **(B)** Increase of caspase 3/7 activity over control in HEK293T cells following stimulation with human recombinant TRAIL and/or Nelson Bay virus (NBV).

In order to determine the influence of TRAIL in HeV-induced apoptotic death of bat cells, we infected both cell lines with HeV and simultaneously treated cells with human recombinant TRAIL. At 24 hpi, bat cells infected with HeV and treated with TRAIL had a significant increase in caspase 3/7 activity compared to control cells (*P* <0.01; Figure 9A). Similar observations were made at 48 hpi and, to a lesser extent, 72 hpi in the bat cells. TRAIL alone also significantly increased caspase 3/7 activity at 24 and 72 hpi, albeit to a lesser extent compared to TRAIL combined with HeV (*P* <0.01). As expected, a significant increase in caspase 3/7 activity was observed in the HeV-infected PaKiT03 cells at 48 and 72 hpi (*P* <0.05; Figure 9A). By contrast, human cells infected with HeV and treated with TRAIL showed no increase in caspase 3/7 activity at any time point (data not shown).

Finally, we set out to demonstrate that human (HEK293T) cells are capable of TRAIL-induced apoptotic cell death following viral infection. For this experiment we utilized the bat orthoreovirus Nelson Bay virus (NBV) [22]. NBV is also known as Pteropine orthoreovirus NB (PRV1NB) [23]. Previous studies have demonstrated that mammalian reoviruses can induce TRAIL-mediated cell death in HEK293T cells. HEK293T cells were infected with NBV at an MOI of 5 and treated with human recombinant TRAIL for 24 hpi. A significant increase in caspase 3/7 activity was observed in these human cells simultaneously infected with NBV and treated with TRAIL compared to human cells infected with NBV and treated with TRAIL alone (*P* <0.01; Figure 9B).

### Up-regulation of *TRAIL* in HeV-infected bat lungs

**Figure 10:**
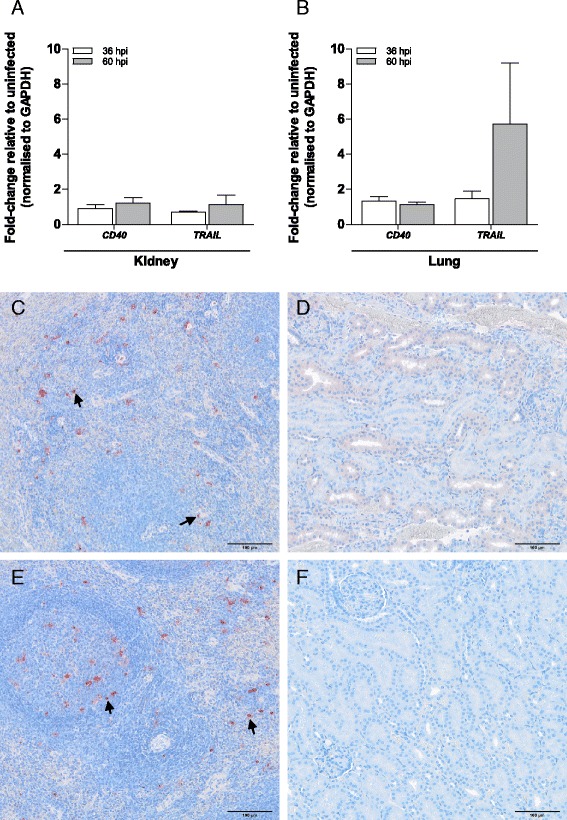
**TRAIL expression and apoptosis in bats** ***in vivo*** **.** Tissue samples were obtained from a previous unpublished HeV infection of *P. alecto* bats (Australian black flying fox). The expression of *CD40* and *TRAIL* mRNA was compared in **(A)** kidney and **(B)** lung and from bats infected with HeV for 36 and 60 hpi compared to uninfected bats. TUNEL staining was also performed on spleen and kidney sections from the experimentally infected and control bats. TUNEL staining of the **(C)** spleen and **(D)** kidney of a representative control bat. Arrows highlight TUNEL-positive cells in the spleen. TUNEL staining from a representative HeV-infected bat (60 hpi) showed no increase in the number of TUNEL-positive cells in the **(E)** spleen and/or **(F)** kidney compared to the control. Scale bar is 100 μm in all panels.

To determine whether *TRAIL* and *CD40* mRNA is induced during HeV infection *in vivo,* we utilized previously collected tissue samples from an experimental infection of the Australian black flying fox. Kidney and lung tissue samples from four experimental HeV-infected and two naïve control bats were obtained from previous, unpublished studies. Bats were infected with HeV for 36 (n = 2) and 60 hours (n = 2). Compared to uninfected animals we observed no increase in *CD40* mRNA expression at either 36 or 60 hpi in either the lung or kidney samples (Figure 10A,B). By contrast, *TRAIL* mRNA was found to be up-regulated approximately five-fold in the lung at 60 hpi (Figure 10B). Because of the biological variation between bats, this increase was not statistically significant. *TRAIL* mRNA was not induced in the lung at 36 hpi or the kidney at either 36 or 60 hpi.

### Apoptotic cell death *in vivo*

Given our previous results *in vitro*, we next set out to investigate whether HeV could induce apoptosis *in vivo* within its natural host, the Australian black flying fox. Formalin-fixed paraffin-embedded tissue sections from the experimental HeV-infected and two naïve control flying foxes were obtained as described above. Colorimetric TUNEL staining was performed on spleen and kidney sections from all bats. Unfortunately the lung sections from this trial were not suitable for TUNEL staining, due to artifacts created during euthanasia. Few apoptotic cells were observed in the kidney in either the HeV-infected (Figure 10C) or control bats (Figure 10D). By contrast, we observed numerous apoptotic cells in the spleen (Figure 10E,F). The number of apoptotic cells within the spleen, however, did not appear to be greater in the HeV-infected bats compared to the naïve controls.

## Discussion

The clinical outcome of humans following HeV infection is significantly different to that of bats. The ability of bats to maintain asymptomatic viral infections whilst the same agent often causes fatal infections in human is a typical feature of bat-borne zoonotic viruses. The molecular mechanisms responsible for this dichotomy remain unknown. Despite the absence of clinical disease, the fact that bats can shed and transmit viruses suggests bats are capable of controlling viruses, but not eliminating them all together. Rapid induction of the innate immune processes has been proposed as one possible mechanism for subduing viral replication in bats [19]. Considering the importance of bats as natural reservoirs for many emerging viruses, elucidating the mechanisms by which bats control viral replication will be invaluable to the field of emerging infectious diseases. In addition, system-wide analysis of human responses to infection only provides a picture of the pathways affected during a pathogenic infection. Ideally we would like to have a system-wide comparison between a pathogenic and non-pathogenic response to the same infectious agent. With this in mind, we compared the transcriptome and proteome responses of immortalized kidney cells from human and bat following HeV infection using our recently developed integrated systems-wide approach.

The PaKiT03 and HEK293T cells examined in this study responded very differently to HeV. Even when only high confidence orthologous transcripts are considered, the transcriptomic and proteomic response was significantly different - despite the similar expression profiles of orthologs at 0 hpi. Human HEK293T cells appeared to show little response at 8 hpi, followed by a largely suppressive response at 24 hpi. PaKiT03 cells, however, demonstrated a strong activation of several immune pathways at 8 and 24 hpi, followed by the induction of extrinsic apoptosis at 24 hpi and beyond.

To our knowledge, this is the first study to examine programmed cell death within a bat species. Apoptosis plays an integral part of the host-pathogen interaction. Induction of the innate immune response often leads to the activation of apoptotic processes, typically through members of the TNF family. We have demonstrated that, following HeV infection, PaKiT03 cells up-regulated components of the TRAIL-mediated apoptosis pathway. By contrast, the HEK293T cells appeared to either down-regulate a number of pro-apoptotic proteins or up-regulate anti-apoptotic components. Induction of TRAIL-mediated apoptosis following viral infection of human cells has been reported previously. In most cases, viral infection induces the expression of the death receptors DR4 and DR5, which in turn sensitizes the cells to TRAIL-mediated apoptosis [24,25]. Infection of human cells with hepatitis C and mammalian reoviruses induces mRNA and protein expression of the functional TRAIL receptors DR4 and DR5 [24,26]. Paramyxoviruses such as human respiratory syncytial virus have also been shown to induce expression of DR5 and DR4 in human lung cells [25]. In the PaKiT03 cells, we observed a greater than two-fold increase in *TNFRSF10A*/*DR4* mRNA expression at 24 hpi. Interrogation of the *P. alecto* genome along with our *de novo* assembled transcriptome suggested that, like mice, bats contain only one functional death receptor. *TNFRSF10A*/*DR4* may therefore act as the sole functional TRAIL receptor in bats.

The expression of TRAIL is regulated through NF-κB transcription factors [27] in response to viral infection. Viruses such as measles and respiratory syncytial virus induce functional TRAIL expression in human cells [25,28]. Here, we observed a significant increase in TRAIL protein expression at 24 hpi in the bat cells. The activation of NF-κB pathways within the bat cells, including the up-regulation of *CD40*, *NFKB2* and *RelB* transcripts, may have contributed to the increased TRAIL protein expression. Surprisingly, we observed no increase in TRAIL mRNA at 8, 24, 48 or 72 hpi within the PaKiT03. This finding suggests that the regulation of TRAIL protein expression possibly occurs post-transcriptionally. By using previously collected lung and kidney tissue samples from HeV-infected bats, we examined the expression of *TRAIL* and *CD40* mRNA in the context of an *in vivo* infection. While no increase in *CD40* mRNA expression was observed in either kidney or lung, we found an approximately five-fold up-regulation of *TRAIL* mRNA in bat lungs infected with HeV for 60 h. Previous, unpublished data from this trial demonstrated that the HeV antigen could only be detected in the lung tissue at 60 hpi through immunohistochemistry (M. Baker, unpublished data). The coordinated up-regulation of *TRAIL* mRNA at the site of HeV infection within the natural host highlights the importance of TRAIL in HeV infection *in vivo*.

The interaction between TRAIL and its functional receptors is crucial for inducing apoptotic cell death through caspase 8 [29]. The treatment of cells with human recombinant TRAIL and subsequent HeV infection demonstrated that HeV sensitizes PaKiT03 cells to TRAIL-mediated apoptosis at 24 hpi. Presumably, this ‘sensitivity’ is achieved through the increased expression of *TNFRSF10A*/*DR4* as discussed above. A similar observation was made by Clarke *et al*. [24], where reovirus infection increased the sensitivity of human cells to apoptosis by increasing the expression of *DR4* and *DR5*. In the present study, HEK293T cells showed no increased susceptibility to TRAIL-mediated apoptosis following HeV infection. Importantly, we showed that this finding is not a general feature of human HEK293T cells. Indeed, when we treated these cells with human recombinant TRAIL and infected them with NBV, we saw an increase in TRAIL-mediated apoptosis (evidenced by increased caspase 3/7 activity). We can therefore conclude that the HEK293T cells are indeed capable of TRAIL-induced cell death, but for unclear reasons this process does not occur in response to HeV.

Pro-apoptotic components down-stream of the death receptors were also induced in the bat cells, including *BBC3*, *BIRC3* and caspase 13. The latter, also known as evolutionarily related interleukin1β converting enzyme, was originally discovered in humans [30], but was later shown to be of bovine origin and is the likely ortholog of human caspase 4 [31]. In the present study, a transcript resembling caspase 13 was induced at 8 and 24 hpi in the bat cells. Previous studies have shown that over-expression of caspase 13 in human HEK293 and MCF7 cells successfully induces apoptosis [30]. Furthermore caspase 13 appeared to be activated by caspase 8 [30], suggesting this enzyme may play a role in death receptor-mediated apoptosis pathways, such as TRAIL.

In principle, the ability of PaKiT03 cells to induce apoptotic cell death following HeV infection could provide a mechanism by which cells can effectively inhibit or subdue viral replication. To determine whether this phenotype was representative of all bat cells we examined HeV-induced apoptosis across three more bat (derived from *P. alecto*) and three more human cell lines. The apoptotic response varied widely between the cell lines. Strong caspase 3/7 responses were observed in HEF and HeLa cells at 24 hpi. The bat fetus cells (PaFeB5) also showed a strong caspase 3/7 response, while the brain (PaBrT03) and lung (PaLuT02) cells were less responsive. While these findings demonstrate that HeV-induced apoptosis is not specific to bat cells, they do highlight the importance of programmed cell death in viral infection more broadly. In agreement, a previous study found NiV partially activates and induces apoptotic cell death of human dendritic cells through up-regulation of caspase 3 and down-regulation of bcl2 [32].

Considering the induction of apoptosis in PaKiT03 cells *in vitro*, along with the up-regulation of *TRAIL* mRNA in HeV-infected bat lung tissues, we attempted to examine the apoptotic response of bats to HeV *in vivo.* TUNEL staining of formalin-fixed paraffin-embedded tissue sections revealed no increase in the proportion of apoptotic cells in either the kidney or spleen of HeV-infected bats compared to uninfected controls. In fact, very few apoptotic cells were observed in the kidney in any sample, while numerous apoptotic cells were found in the spleen. Unfortunately, the lung sections (where we might have expected to see the most striking differences) from this trial were not suitable for TUNEL staining, due to artifacts created during euthanasia. While this experiment could not definitively demonstrate HeV-induced apoptosis *in vivo* in bats, future examination of tissues from other HeV model species such as ferret and mice - where a more pronounced viral load is achieved - may help clarify the influence of apoptosis *in vivo*.

We cannot exclude the possibility that the subtle differences in viral transcription between the bat and human cells are influencing the host response. Viruses utilize a number of strategies for counteracting immune and apoptotic signals. Previous studies have demonstrated that paramyxoviruses can use the accessory proteins from the P/V/C gene to inhibit apoptosis [33,34]. The V protein of mumps virus is also known to block IFN expression [35]. When we compared the HeV transcription profile between the cell lines, there appeared to be fewer transcripts for the P/V/W region in human cells compare to bat cells. Considering the important role of the V protein in immune evasion, this transcriptional variation may influence the host response, particularly in regard to IFN production. Phenotypically, we also observed differences in the cytopathic effect between human and bat cells following HeV infection at 24 hpi. Extensive syncytia were observed in the human but not bat cells. Cell fusion and syncytia are mediated by the HeV-F protein. It is therefore interesting to note that we observed higher abundance of F transcript in the human cells compared to bat cells. Differences in the abundance of F transcript may therefore influence the difference in cytopathic effect observed.

The induction of IFN-β1 and down-stream ISGs in human cells was unexpected. A number of transfection studies have demonstrated that the henipavirus P gene products (P, V, W and C) can inhibit both the IFN induction and IFN signaling pathways in human cell lines (including HEK293T and 2fTGH) [36,37]. More recent studies on HEK293T cells, however, have shown that, while productive henipavirus infection can effectively block IFN production after 3 hpi, it only partially blocks IFN signaling, as measured by the expression of ISGs at 24 hpi [38]. Similar studies on a range of *P. alecto* cell types, including lung (PaLuT02), primary kidney (PaKi) and fetus (PaFe and PaFeT) [39], demonstrated that both IFN production and signaling were blocked by henipavirus infection at 3 and 24 hpi, respectively [40]. By contrast, our findings demonstrate that in human HEK293T cells IFN production and signaling occur at 24 hpi, thus suggesting the host is capable of overcoming the antagonistic effects of HeV at this later time point. It is worth noting that interferon regulatory factor 3 (IRF3), an important transcription factor responsible for IFN-β induction, was down-regulated at the protein level at 24 hpi in human cells. This finding is somewhat at odds with the induction of IFN-β1 at 24 hpi in human cells. NiV has been shown to block IFN production through the accessory V and W proteins by targeting IRF3 [41]. While HeV may use a similar mechanism, a two-fold reduction in IRF3, as observed in this study, does not appear sufficient to completely abolish IFN production in human cells. IFN antiviral response is also mediated to some degree by the Ras/MAPK pathways. Studies of persistent Ebola virus (Mayinga strain) infection of bat lung fibroblasts found that activation of the Ras/MAPK pathway, which antagonizes the IFN response, was required for productive infection [13]. In the present study, we found many of the down-regulated transcripts/proteins in the human cells were mapped to MAPK pathways. Inactivation of these pathways may have contributed to the increased IFN response observed in human cells at 24 hpi.

It should be acknowledged that this study, although comprehensive, examined only one cell type from both bats and humans. The major differences in response between the bat and human cells may to some degree simply reflect differences in cell type. In any mammal, it is highly likely that not all cell types will respond to viral infection in a similar fashion. Furthermore, mechanisms deduced from cell lines may not reflect what occurs *in vivo*. With this in mind, we stress that caution must be taken when extending conclusions from *in vitro* studies (such as ours) to other cell types, and more generally *in vivo* models. However, *in vitro* studies provide significant insight into host mechanisms at the molecular level, ultimately generating numerous hypotheses that can be investigated within appropriate models *in vivo*. Indeed, *in vitro* studies are necessary (at least in the first instance) when examining a highly pathogenic virus (such as HeV) that requires high levels of bio-containment.

Turning to the many novel genes and proteins identified, that we uncovered a great many new genes that are also positively identified by liquid chromatography tandem mass spectrometry (LC-MS/MS) re-enforces the power of our PIT-based approach to comprehensively identify, annotate and quantitate changes in human and non-model host species. This is the first time a single virus has been comprehensively analyzed by RNAseq and high-throughput quantitative proteomics in cells lines from two species. For the first time, we can compare at a systems level, the response between a resistant reservoir host (bat) and a susceptible spillover host (human). By identifying the crucial cellular pathways whose differential activation correlates with outcome, we open new opportunities for therapeutic intervention, through testing the many pathway-specific licensed drugs already available. Moreover, our approach enables side-by-side comparison of any mammal with humans to determine how similar or divergent two species are when they are infected with a virus or exposed to a drug (or both). In principle, this could guide better selection of animal models and improve our ability to predict how humans will respond to treatments developed in animal models. Finally, our dataset provides transcriptomic and proteomic evidence of approximately 5,900 genes and proteins. This includes evidence for nearly several hundred genes and proteins not listed in the UniProt dataset for *P. alecto*. This represents a major leap forward in our understanding of the genetic content of bats - significant natural hosts of current and potentially future zoonotic agents.

## Conclusion

By application of PIT analysis, we have shown that PaKiT03 and HEK293T cells respond significantly differently to HeV at both the mRNA and protein level. Human HEK293T cells demonstrated little early response followed by a global suppression of mRNA and protein abundance. Bat PaKiT03 cells, by contrast, demonstrated a robust innate immune response, which led to the execution of extrinsic TRAIL apoptosis pathways. At 48 and 72 hpi, PaKiT03 cells, but not HEK293T cells, demonstrated a significant increase in apoptotic cell death. However, when further cell lines from bats and humans were examined, we found that at least some human lines (HEF and HeLa) were susceptible to HeV-mediated apoptosis. Moreover, examination of tissue samples from HeV-infected bats failed to reveal widespread apoptosis at least for the spleen and kidneys. Notwithstanding this, we have shown how to compare the response of two distinct species to the same infectious agent using RNAseq and high-throughput quantitative proteomics. We have used this analysis to highlight the potential to identify and test pathways that in principle could have a significant bearing on the outcome of disease. We believe this approach will enable the identification and investigation of pathways that contribute at a molecular level to the devastating differential pathogenicity of zoonotic bat viruses when they infect humans.

## Methods

### Cell culture and SILAC

The *P. alecto* immortalized kidney derived cell line PaKiT03 [39] and the human immortalized embryonic kidney cell line HEK293T were predominantly used in this study. Cells were cultured in either Dulbecco’s modified Eagle’s medium (DMEM)/F12 (PaKiT03) or DMEM (HEK293T) media (Pierce, Rockford, USA) containing 10% (v/v) dialyzed fetal calf serum (FCS), and 10 mM HEPES. To achieve isotope incorporation cells were cultured in either ^12^C/^14^ N lysine and arginine (designated ‘light’), ^13^C/^14^ N lysine and arginine (designated ‘mid’) or ^13^C/^15^ N lysine and arginine (designated ‘heavy’). Cells were cultured for at least 10 doublings.

Additional bat cell lines utilized in this study included *P. alecto* immortalized brain (PaBrT03), fetus (PaFeB5) and lung (PaLuT02) cells [39]. All bat cells were cultured in DMEM/F12 containing 10% FCS. Additional human cells utilized in this study include A549, HEF and HeLa cells. The A549 and HEF cells were cultured in DMEM containing 10% FCS. HeLa cells were cultured in Eagle's minimal essential medium containing 10% FCS.

### Virus infection for SILAC cells

Approximately 2 × 10^7^ PaKiT03 and HEK293T cells were either mock infected (light), or infected with HeV/Australia/Horse/1994/Hendra strain for 8 h (mid) or 24 h (heavy) at an MOI of 10. The experiment was conducted in duplicate in T75cm^2^ flasks. Cells were harvested by trypsinization and split in two. One half was resuspended in 2% SDS-NuPAGE reducing buffer (Life Technologies, Carlsbad, USA) and boiled for 5 min at 100°C. The remainder was resuspended in RLT buffer (Qiagen, Limburg, Netherlands) containing 1% β-mecaptoethanol.

### Immunofluorescence microscopy

SILAC-labeled PaKiT03 and HEK293T cells were seeded onto 13 mm diameter coverslips in 24-well plates. Cells were either mock (light cells) or infected for 8 h (mid) or 24 h (heavy) with HeV/Australia/Horse/1994/Hendra strain at an MOI of 10. Media was removed and cells were fixed in 4% paraformaldehyde (w/v) in phosphate-buffered saline (PBS) for 1 h. Coverslips were then washed three times in PBS, permeabilized with 0.1% (w/v) Triton X-100 for 10 min, blocked with 0.5% (w/v) bovine serum albumin (BSA) for 30 min, and incubated with anti-N protein (Australian Animal Health Laboratory) diluted 1:2,000 in 0.5% (w/v) BSA for 1 h. Cells were washed three times with PBS and treated with a Alexa 488-labeled goat anti-rabbit antibody diluted 1:200 (Life Technologies) in 0.5% (w/v) BSA for 1 h. Finally, cells were washed twice with PBS, once with H_2_O, and stained with 4′,6-diamidino-2-phenylindole dihydrochloride for 10 min. Coverslips were washed in PBS, mounted in Vectashield (Vector Labs, Burlingame, USA) and imaged with a Leica SP5 confocal microscope.

### RNA isolation and sequencing

Total RNA was isolated from cells in RLT buffer using the RNAeasy kit (Qiagen) with DNase I treatment as per the manufacturer’s instructions. The quality and quantity of RNA was assessed for all samples using a Bioanalyser (Agilent, Santa Carla, USA). mRNA was sequenced as 100 base pair paired-end reads across a single lane on a HiSeq 2000 (Illumina, San Diego, USA). Resulting reads were trimmed for adapters and quality assessed using FastQC. Reads have been deposited in the NCBI Sequence Read Archive and assigned the accession [SRP044809].

### Gene Ontology and KEGG enrichment

Official gene identifications (IDs) were retrieved for all significantly differentially expressed transcripts and proteins. Gene IDs for transcripts and proteins that were significantly up-regulated at one or more time points were combined into a single list. A list of significantly down-regulated gene IDs was also compiled. Biological process GO enrichment was performed separately on the up- and down-regulated transcript and protein lists using the unranked target and background analysis in GOrilla [42]. Background gene lists were compiled by retrieving all gene IDs from either the human (hg19) or *P. alecto* genome. Enriched GO categories were compiled into Excel spreadsheets and visualized using REVIGO [43]. The up- and down-regulated transcript and protein lists were also used to interrogate KEGG pathways using the standard KEGG Mapper tool [44].

### Transcriptome assemblies and differential expression analysis

Raw reads from each species were first used to assemble a *de novo* transcriptome on the University of Bristol high performance computing facility, BlueCrystal, using Trinity [45,46]. Next, for each of the samples, reads were independently mapped against the *P. alecto* [GenBank:GCF_000325575.1] or human (hg19 in Ensembl) genome with Bowtie (version 0.12.7) and TopHat (version 1.3.2) [47]. Using the alignment outputs, transcripts were assembled for each sample and merged into a single transcriptome for PaKiT03 and HEK293T using Cufflinks and Cuffmerge (version 1.2.1) [48]. Both Trinity and Cufflinks-assembled transcriptomes were translated as previously described [21] using the ‘getorf’ facility in EMBOSS and ORFs less than 200 nucleotides were removed.

To understand changes in transcript abundance, reads from each time point were mapped separately against the Trinity-generated transcriptome using Bowtie2 (version 2.1.0). Resulting SAM files were converted to BAM, sorted, indexed, and the number of reads mapping to each Trinity transcript was compiled using SAMtools (version 0.1.18). Differential expression analysis was conducted with DESeq (version 1.14.0). Count data was normalized and pairwise comparisons between time points calculated as described previously [49]. Transcripts with adjusted *P*-values <0.05 were considered significant.

### Quantitative proteomics

The three SDS boiled protein samples were combined in a 1:1:1 ratio, separated by SDS-PAGE and analyzed by LC-MS/MS. The gel lane was cut into 20 slices, and each slice was subjected to in-gel tryptic digestion and the samples processed as described previously [21]. In addition, the samples from PakTi03 cells were run again (another 20 slices) as a technical repeat to maximize our data return for this species.

The raw data files were processed and quantified using MaxQuant and searched against the three databases described below. Peptide precursor mass tolerance was set at 10 ppm, and MS/MS tolerance was set at 0.8 Da. Search criteria included carbamidomethylation of cysteine (+57.0214) as a fixed modification and oxidation of methionine (+15.9949) and appropriate SILAC labels (^13^C_6_-lysine, ^13^C_6_-arginine for mid-labeled cells and ^13^C6, ^15^ N_2_-lysine, ^13^C_6_, ^15^ N_4_-arginine for heavy-labeled cells as variable modifications). Searches were performed with full tryptic digestion, and a maximum of two missed cleavages was allowed. The reverse database search option was enabled, and all peptide data were filtered to satisfy a false discovery rate of 1%. All raw proteomics data has been deposited into the PRIDE (PRoteomics IDEntifications) database and assigned the accession number [PXD001165].

The lists of proteins derived from a Trinity- or Cufflinks-based assembly of mRNA were searched sequentially on their own to determine which ORFs might be real (that is, at least one peptide identified). From this analysis we obtained a short list of ORFs that had been identified and combined these with the official UniProt lists to create a combined list of proteins from UniProt, from the Trinity transcripts and from the Cufflinks-assembled transcripts. To identify the maximum number of peptides, mass spectra were searched against a combination of three protein databases: UniProt-derived lists of proteins for HeV and either *H. sapiens* or *P. alecto*; ORFs identified as real from the Cufflinks-derived mRNA; and ORFs identified as real from the *de novo* assembled Trinity-derived mRNA. Peptides were assigned by MaxQuant into ‘proteinGroups’ that contain one or more individual protein sequences (Additional files 7 and 8). In most cases peptides were assigned to a proteinGroup that contained a protein sequence from all three databases (Additional file 9). For the PaKiT03 cells the greatest number of proteinGroups was identified against the Trinity *de novo* assembled transcript database. By contrast, for the HEK293T cells the greatest number of proteinGroups was identified against the UniProt human proteins list (Additional file 9). Differential expression of the proteinGroups was calculated from the combined peptide abundance ratio of mid to light (8 hpi:0 hpi) and heavy to light (24 hpi:0 hpi). A threshold of ≥ two-fold change was employed. That is, protenGroups with SILAC ratios ≥2 or ≤0.5 were considered significant.

### Comparison between human and *Pteropus* datasets

To identify orthologs shared between bats and humans we used the Trinity-derived lists of transcripts. ORFs (from start codon to stop codon) were translated in all frames from the Trinity-derived transcripts and ORFs over 66 amino acids long were compiled into a table together with the transcript they came from. This list of ORFs was searched using BLAST to find the closest homolog in the human UniProt list. This process was repeated for both the human and the bat list of transcripts. Finally, for each entry in the human UniProt list of proteins we selected the best match from the Trinity-derived ORFs for each species. In this way we were able to generate a master list of transcripts and ORFs that were the best matches to the human UniProt list of proteins. This approach allowed us to connect orthologous genes and ORFs from the human Trinity-derived transcripts to the *P. alecto* Trinity-derived transcripts and ORFs. We were then able to designate the *P. alecto* genes and proteins with their nearest human homologs, enabling a pathways analysis based on the assumption that all the identified *P. alecto* genes and proteins had direct human homologs (Additional file 4). Alongside this analysis, we examined the list of *P. alecto* Trinity-derived ORFs that had been identified as being real on the basis that at least one peptide from each ORF had been detected by the MaxQuant analysis. In essence, each transcript that generated an ORF identified by MaxQuant was compiled into a list of transcripts, ORF and peptide evidence for each ORF. We then used sequential BLAST searches to identify each ORF by searching against the *P. alecto* proteome and the human proteome (Additional file 10). We separated out any ORF which had a less than 60% identity to either official proteome list and searched only those against the non-redundant protein lists, again using BLAST (Additional file 11).

### Bat tissue samples

Tissue samples from *P. alecto* were obtained from a previous experimental HeV infection at the Australian Animal Health Laboratory. Seven age-matched Australian black flying foxes were each intranasally infected with 27,300 Tissue culture infective dose (TCID50)/ml of HeV (Redlands strain). Two uninfected bats served as controls. Animals were euthanized at 12 (n = 3), 36 (n = 2) and 60 (n = 2) hpi, and tissues dissected and stored. RNA was extracted (as described above) from lung and kidney for the 36 hpi, 60 hpi and control bats. Lung, kidney and spleen tissues were formalin fixed and paraffin embedded for all bats. All animal experimentation was approved by the Commonwealth Scientific and Industrial Research Organisation-Australian Animal Health Laboratory Animal Ethics Committee (protocol AEC1558).

### Real-time PCR

Differential gene expression was assessed using reverse transcriptase real-time PCR. Primers were designed using Primer3 (Additional file 12). Reaction parameters were identical for all genes. Briefly, 5 μg of total RNA was reverse transcribed using Superscript III (Life Technologies) primed with oligo-dT as per the manufacturer’s instructions. Triplicate SYBR green real-time PCR reactions were performed in a 25 μl reaction, containing 1X EXPRESS SYBR green master mix (Life Technologies), 200 nM forward and reverse primer, and 20 ng template. Cycling parameters were 95°C for 10 min, then 40 cycles of 95°C for 30 s, 55°C for 30 s and 72°C for 1 min, followed by melt curve analysis. Differential expression was calculated relative to uninfected T0, normalized to GAPDH using the relative expression software tool REST [50].

### Western blotting

Protein quantity was determined for SDS boiled lysates using the EZQ Protein Quantitation Kit (Life Technologies) following the manufacturer’s instructions. For each sample a total of 10 μg of protein lysate was subjected to SDS-PAGE and transferred to a polyvinylidene difluoride membrane. Membranes were blocked with 5% skim milk powder for 1 h. Membranes were washed three times (10 min each) with Tris-buffered saline/Tween 20 (TBST), and then incubated for 1 h with primary antibody diluted in TBST. The following primary rabbit antibodies were used: CD40 (1:5,000, Thermo Fisher, Waltham, USA), A20 (1:1,000, Cell Signaling, Danvers, USA) and β2-tubulin (1:2,000, Cell Signaling). Membranes were washed three times with TBST and then incubated for 1 h with goat anti-rabbit horseradish peroxidase-conjugated secondary antibody diluted in TBST. Membranes were finally washed twice with TBST, once with TBS and developed with ECL-Plus chemiluminescent substrate (Thermo Fisher) as per the manufacturer’s instructions. Membranes were scanned at 473 nm on a Typhon FLA9000 gel imaging scanner (GE Healthcare, Little Chalfont, UK).

### Apoptosis and viability assay

The Caspase-Glo 3/7 Assay (Promega, Madison, USA) was used to determine the activity of caspase 3/7. Cell viability was determined using the CellTiter-Glo Luminescent Cell Viability Assay (Promega). For both assays, approximately, 30,000 cells/well were seeded into white 96-well plates. Cells were infected with HeV or NBV [22] for 24, 48 and 72 h at an MOI of approximately 5. Both assays were performed exactly as per the manufacturer’s instructions, in triplicate. TRAIL stimulation with performed using Super*Killer*TRAIL (AdipoGen, San Diego, USA) at 500 ng/ml diluted in media. One-way analysis of variance was used to compare the caspase 3/7 luminescence over the control.

### TUNEL staining

TUNEL was used to identify apoptotic PaKiT03 and HEK293T cells following viral infection. Approximately 1.5 × 10^5^ cells were seeded onto 15 mm diameter coverslips in individual wells of a 24-well plate. Cells were either mock or infected for 8, 24 or 48 h with HeV/1994/Australia strain at an MOI of 5. Media was removed and cells were fixed in 4% (w/v) paraformaldehyde in PBS for 1 h. Cells were then gently washed three times in PBS. Staining was performed using a Click-iT TUNEL Alexa Fluor® 488 kit as per the manufacturer’s instructions (Life Technologies). Optional immunofluorescence was performed against the HeV-N protein as described above except a secondary Alexa Fluor 568 Goat Anti-Rabbit antibody (Life Technologies) was used.

Colormetric TUNEL staining was performed on Australian black flying fox spleen and kidney tissue sections from a previous unpublished experimental HeV infection. Following routine histological processing and paraffin embedding, blocks were sectioned at 4 μm on to positively charged slides and placed in a 37°C oven to dry. Sections were placed in a 60°C incubator for 10 min prior to de-waxing and rehydration through a series of graded alcohols to water. The TUNEL staining for apoptotic cells was carried out as per the manufacturer’s instructions for tissue sections using the DeadEnd Colorimetric TUNEL System (Promega) with the exception of a substitution of 3-amino-9-ethylcarbazol for 3,3′-diaminobenzidine as the chromogen. The sections were subsequently counterstained with Lillie-Mayer’s hematoxylin to enable visualization of the morphological structure of the tissue.

## Additional files

Additional file 1
**List of differentially expressed Trinity transcripts and proteins at 8 and 24 hpi in PaKiT03 (sheet one) and HEK293T cells (sheet two).** Top BLAST hit against SwissProt is given. Additional file 2
**Full list of Trinity transcript DEseq and SILAC protein expression statistics in PaKiT03.**
Additional file 3
**Full list of Trinity transcript DEseq and SILAC protein expression statistics in HEK293T.**
Additional file 4
**Ortholog identification of between human and bat.** The most similar ortholog between bat and human is given. Additional file 5
**Enriched GO terms from combined transcript and protein lists from GOrilla analysis.**
Additional file 6
**Enriched KEGG pathways from combined transcript and protein lists.**
Additional file 7
**All proteinGroups identified in PaKiT03 cells through interrogation of UniProt, Cufflinks and Trinity proteome databases.**
Additional file 8
**All proteinGroups identified in HEK293T cells through interrogation of UniProt, Cufflinks and Trinity proteome databases.**
Additional file 9
**Mass spectra were searched against three protein databases: UniProt** ***(H. sapiens*** **or** ***P. alecto)*** **, translated Trinity transcriptome and translated Cufflinks transcriptome for** **(A)** **PaKiT03 and** **(B)** **HEK293T.** The number of proteinGroups identified and shared between the databases is given. Additional file 10
**BLAST analysis of bat Trinity transcripts where one or more peptides were identified.** BLAST was used against the *P. alecto* and human proteomes (UniProt). Additional file 11
**BLAST analysis against NCBI non-redundant database of Trinity transcripts that were <60% similar to both the** ***P. alecto*** **and human proteomes.**
Additional file 12
**Primer sequences used for this study.**

## Abbreviations

BLAST: Basic Local Alignment Search Tool
BSA: bovine serum albumin
DMEM: Dulbecco’s modified Eagle’s medium
FCS: fetal calf serum
GO: Gene Ontology
HeV: Hendra virus
hpi: hours post infection
IFN: interferon
ISG: interferon-stimulated gene
KEGG: Kyoto Encyclopedia of Genes and Genomes
LC-MS/MS: liquid chromatography tandem mass spectrometry
MOI: multiplicity of infection
NBV: Nelson Bay virus
NF-κB: nuclear factor kappa-light-chain-enhancer of activated B cells
NiV: Nipah virus
ORF: open reading frame
PARP1: poly (ADP-ribose) polymerase 1
PBS: phosphate-buffered saline
PCR: polymerase chain reaction
PIT: proteomics informed by transcriptomics
SDS-PAGE: sodium dodecyl sulfate polyacrylamide gel electrophoresis
SILAC: stable isotope labeling of amino acid in cell culture
TBST: tris-buffered saline and Tween 20
TNF: tumor necrosis factor
TRAIL: tumor necrosis factor-related apoptosis inducing ligand
TUNEL: terminal deoxynucleotidyl transferase dUTP nick end labeling

## References

[1] Jones KE, Patel NG, Levy MA, Storeygard A, Balk D, Gittleman JL, Daszak P (2008). Global trends in emerging infectious diseases. Nature.

[2] Luis AD, Hayman DTS, O'Shea TJ, Cryan PM, Gilbert AT, Pulliam JRC, Mills JN, Timonin ME, Willis CKR, Cunningham AA, Fooks AR, Rupprecht CE, Wood JLN, Webb CT (2013). A comparison of bats and rodents as reservoirs of zoonotic viruses: are bats special?. Proc R Soc Lond B Biol Sci.

[3] O’Sullivan JD, Allworth AM, Paterson DL, Snow TM, Boots R, Gleeson LJ, Gould AR, Hyatt AD, Bradfield J (1997). Fatal encephalitis due to novel paramyxovirus transmitted from horses. Lancet.

[4] Selvey LA, Wells RM, Mccormack JG, Ansford AJ, Murray K, Rogers RJ, Lavercombe PS, Selleck P, Sheridan JW (1995). Infection of humans and horses by a newly described morbillivirus. Med J Aust.

[5] Chua KB, Goh KJ, Wong KT, Kamarulzaman A, Tan PSK, Ksiazek TG, Zaki SR, Paul G, Lam SK, Tan CT (1999). Fatal encephalitis due to Nipah virus among pig-farmers in Malaysia. Lancet.

[6] Lau SKP, Woo PCY, Li KSM, Huang Y, Tsoi HW, Wong BHL, Wong SSY, Leung SY, Chan KH, Yuen KY (2005). Severe acute respiratory syndrome coronavirus-like virus in Chinese horseshoe bats. Proc Natl Acad Sci U S A.

[7] Li WD, Shi ZL, Yu M, Ren WZ, Smith C, Epstein JH, Wang HZ, Crameri G, Hu ZH, Zhang HJ, Zhang JH, McEachern J, Field H, Daszak P, Eaton BT, Zhang SY, Wang LF (2005). Bats are natural reservoirs of SARS-like coronaviruses. Science.

[8] Leroy EM, Kumulungui B, Pourrut X, Rouquet P, Hassanin A, Yaba P, Delicat A, Paweska JT, Gonzalez JP, Swanepoel R (2005). Fruit bats as reservoirs of Ebola virus. Nature.

[9] Corman VM, Ithete NL, Richards LR, Schoeman MC, Preiser W, Drosten C, Drexler JF: **Rooting the phylogenetic tree of MERS-Coronavirus by characterization of a conspecific virus from an African Bat.** *J Virol* 2014. in press. 10.1128/JVI.01498-14 PMC4178802 25031349

[10] Ithete NL, Stoffberg S, Corman VM, Cottontail VM, Richards LR, Schoeman MC, Drosten C, Drexler JF, Preiser W (2013). Close relative of human Middle East respiratory syndrome coronavirus in bat, South Africa. Emerg Infect Dis.

[11] Field H, McCall B, Barrett J (1999). Australian bat lyssavirus infection in a captive juvenile black flying fox. Emerg Infect Dis.

[12] McColl KA, Chamberlain T, Lunt RA, Newberry KM, Middleton D, Westbury HA (2002). Pathogenesis studies with Australian bat lyssavirus in grey-headed flying foxes (*Pteropus poliocephalus*). Aust Vet J.

[13] Strong JE, Wong G, Jones SE, Grolla A, Theriault S, Kobinger GP, Feldmann H (2008). Stimulation of Ebola virus production from persistent infection through activation of the Ras/MAPK pathway. Proc Natl Acad Sci U S A.

[14] Zhang GJ, Cowled C, Shi ZL, Huang ZY, Bishop-Lilly KA, Fang XD, Wynne JW, Xiong ZQ, Baker ML, Zhao W, Tachedjian M, Zhu YB, Zhou P, Jiang XT, Ng J, Yang L, Wu LJ, Xiao J, Feng Y, Chen YX, Sun XQ, Zhang Y, Marsh GA, Crameri G, Broder CC, Frey KG, Wang LF, Wang J (2013). Comparative analysis of bat genomes provides insight into the evolution of flight and immunity. Science.

[15] Murray K, Selleck P, Hooper P, Hyatt A, Gould A, Gleeson L, Westbury H, Hiley L, Selvey L, Rodwell B, Ketterer P (1995). A morbillivirus that caused fatal disease in horses and humans. Science.

[16] Lindblad-Toh K, Garber M, Zuk O, Lin MF, Parker BJ, Washietl S, Kheradpour P, Ernst J, Jordan G, Mauceli E, Ward LD, Lowe CB, Holloway AK, Clamp M, Gnerre S, Alfoldi J, Beal K, Chang J, Clawson H, Cuff J, Di Palma F, Fitzgerald S, Flicek P, Guttman M, Hubisz MJ, Jaffe DB, Jungreis I, Kent WJ, Kostka D, Lara M (2011). A high-resolution map of human evolutionary constraint using 29 mammals. Nature.

[17] Parker J, Tsagkogeorga G, Cotton JA, Liu Y, Provero P, Stupka E, Rossiter SJ (2013). Genome-wide signatures of convergent evolution in echolocating mammals. Nature.

[18] Seim I, Fang XD, Xiong ZQ, Lobanov AV, Huang ZY, Ma SM, Feng Y, Turanov AA, Zhu YB, Lenz TL, Gerashchenko MV, Fan DD, Yim SH, Yao XM, Jordan D, Xiong YQ, Ma Y, Lyapunov AN, Chen GX, Kulakova OI, Sun YD, Lee SG, Bronson RT, Moskalev AA, Sunyaev SR, Zhang GJ, Krogh A, Wang J, Gladyshev VN (2013). Genome analysis reveals insights into physiology and longevity of the Brandt’s bat *Myotis brandtii*. Nat Commun.

[19] Papenfuss AT, Baker ML, Feng ZP, Tachedjian M, Crameri G, Cowled C, Ng J, Janardhana V, Field HE, Wang LF (2012). The immune gene repertoire of an important viral reservoir, the Australian black flying fox. BMC Genomics.

[20] Shaw TI, Srivastava A, Chou WC, Liu L, Hawkinson A, Glenn TC, Adams R, Schountz T (2012). Transcriptome sequencing and annotation for the Jamaican fruit bat (*Artibeus jamaicensis*). PLoS One.

[21] Evans VC, Barker G, Heesom KJ, Fan J, Bessant C, Matthews DA (2012). De novo derivation of proteomes from transcriptomes for transcript and protein identification. Nat Methods.

[22] Gard GP, Marshall ID (1973). Nelson-Bay Virus - novel reovirus. Arch Gesamte Virusforsch.

[23] Voon K, Chua KB, Yu M, Crameri G, Barr JA, Malik Y, Wang LF (2011). Evolutionary relationship of the L- and M-class genome segments of bat-borne fusogenic orthoreoviruses in Malaysia and Australia. J Gen Virol.

[24] Clarke P, Meintzer SM, Gibson S, Widmann C, Garrington TP, Johnson GL, Tyler KL (2000). Reovirus-induced apoptosis is mediated by TRAIL. J Virol.

[25] Kotelkin A, Prikhod'ko EA, Cohen JI, Collins PL, Bukreyev A (2003). Respiratory syncytial virus infection sensitizes cells to apoptosis mediated by tumor necrosis factor-related apoptosis-inducing ligand. J Virol.

[26] Deng ZF, Yan HJ, Hu JJ, Zhang SW, Peng P, Liu QZ, Guo DY (2012). Hepatitis C virus sensitizes host cells to TRAIL-induced apoptosis by up-regulating DR4 and DR5 via a MEK1-dependent pathway. PLoS One.

[27] Baetu TM, Kwon H, Sharma S, Grandvaux N, Hiscott J (2001). Disruption of NF-kappa B signaling reveals a novel role for NF-kappa B in the regulation of TNF-related apoptosis-inducing ligand expression. J Immunol.

[28] Vidalain PO, Azocar O, Lamouille B, Astier A, Rabourdin-Combe C, Servet-Delprat C (2000). Measles virus induces functional TRAIL production by human dendritic cells. J Virol.

[29] Kassis R, Larrous F, Estaquier J, Bourhy H (2004). Lyssavirus matrix protein induces a poptosis by a TRAIL-dependent mechanism involving caspase-8 activation. J Virol.

[30] Humke EW, Ni J, Dixit VM (1998). ERICE, a novel FLICE-activatable caspase. J Biol Chem.

[31] Koenig U, Eckhart L, Tschachler E (2001). Evidence that caspase-13 is not a human but a bovine gene. Biochem Biophys Res Commun.

[32] Gupta M, Lo MK, Spiropoulou CF (2013). Activation and cell death in human dendritic cells infected with Nipah virus. Virology.

[33] Chambers R, Takimoto T (2009). Antagonism of innate immunity by paramyxovirus accessory proteins. Viruses-Basel.

[34] Sun M, Rothermel TA, Shuman L, Aligo JA, Xu SB, Lin Y, Lamb RA, He B (2004). Conserved cysteine-rich domain of paramyxovirus simian virus 5 V protein plays an important role in blocking apoptosis. J Virol.

[35] Yang ZH (1997). PAML: a program package for phylogenetic analysis by maximum likelihood. Comput Appl Biosci.

[36] Rodriguez JJ, Horvath CM (2004). Host evasion by emerging paramyxoviruses: Hendra virus and Nipah virus V proteins inhibit interferon signaling. Viral Immunol.

[37] Rodriguez JJ, Parisien JP, Horvath CM (2002). Nipah virus V protein evades alpha and gamma interferons by preventing STAT1 and STAT2 activation and nuclear accumulation. J Virol.

[38] Virtue ER, Marsh GA, Wang LF (2011). Interferon signaling remains functional during henipavirus infection of human cell lines. J Virol.

[39] Crameri G, Todd S, Grimley S, McEachern JA, Marsh GA, Smith C, Tachedjian M, De Jong C, Virtue ER, Yu M, Bulach D, Liu JP, Michalski WP, Middleton D, Field HE, Wang LF (2009). Establishment, immortalisation and characterisation of Pteropid bat cell lines. PLoS One.

[40] Virtue ER, Marsh GA, Baker ML, Wang LF (2011). Interferon production and signaling pathways are antagonized during henipavirus infection of fruit bat cell lines. PLoS One.

[41] Shaw ML, Cardenas WB, Zamarin D, Palese P, Basler CF (2005). Nuclear localization of the Nipah virus W protein allows for inhibition of both virus- and toll-like receptor 3-triggered signaling pathways. J Virol.

[42] Eden E, Navon R, Steinfeld I, Lipson D, Yakhini Z (2009). GOrilla: a tool for discovery and visualization of enriched GO terms in ranked gene lists. BMC Bioinformatics.

[43] Supek F, Bosnjak M, Skunca N, Smuc T (2011). REVIGO summarizes and visualizes long lists of gene ontology terms. PLoS One.

[44] Kanehisa M, Goto S (2000). KEGG: Kyoto Encyclopedia of Genes and Genomes. Nucleic Acids Res.

[45] Grabherr MG, Haas BJ, Yassour M, Levin JZ, Thompson DA, Amit I, Adiconis X, Fan L, Raychowdhury R, Zeng QD, Chen ZH, Mauceli E, Hacohen N, Gnirke A, Rhind N, di Palma F, Birren BW, Nusbaum C, Lindblad-Toh K, Friedman N, Regev A (2011). Full-length transcriptome assembly from RNA-Seq data without a reference genome. Nat Biotechnol.

[46] Haas BJ, Papanicolaou A, Yassour M, Grabherr M, Blood PD, Bowden J, Couger MB, Eccles D, Li B, Lieber M, MacManes MD, Ott M, Orvis J, Pochet N, Strozzi F, Weeks N, Westerman R, William T, Dewey CN, Henschel R, Leduc RD, Friedman N, Regev A (2013). De novo transcript sequence reconstruction from RNA-seq using the Trinity platform for reference generation and analysis. Nat Protoc.

[47] Trapnell C, Pachter L, Salzberg SL (2009). TopHat: discovering splice junctions with RNA-Seq. Bioinformatics.

[48] Trapnell C, Roberts A, Goff L, Pertea G, Kim D, Kelley DR, Pimentel H, Salzberg SL, Rinn JL, Pachter L (2012). Differential gene and transcript expression analysis of RNA-seq experiments with TopHat and Cufflinks. Nat Protoc.

[49] Anders S, Huber W (2010). Differential expression analysis for sequence count data. Genome Biol.

[50] Pfaffl MW, Horgan GW, Dempfle L (2002). Relative expression software tool (REST) for group-wise comparison and statistical analysis of relative expression results in real-time PCR. Nucleic Acids Res.

