# Supplementary figures and images for: Proteomics informed by transcriptomics reveals Hendra virus sensitizes bat cells to TRAIL-mediated apoptosis
Source: Genome Biol. 2014 Nov 15;15(11):532. doi: 10.1186/s13059-014-0532-x (PMC4269970; doi:10.1186/s13059-014-0532-x)

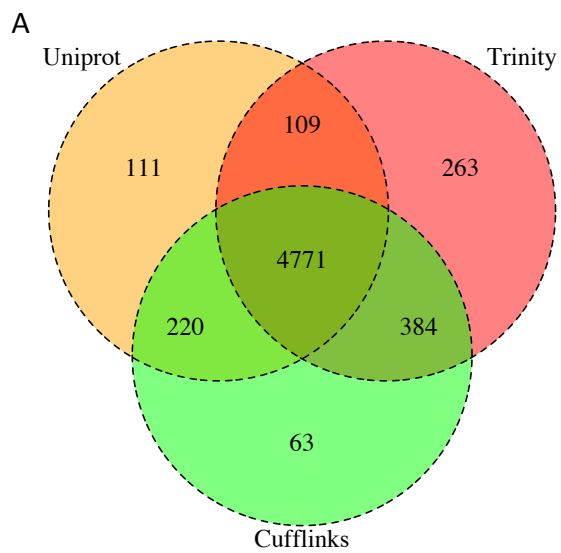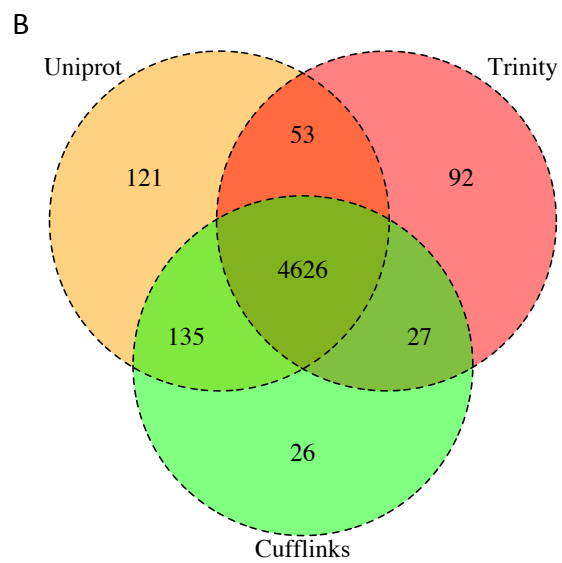

Supplement: Additional file 9 — Mass spectra were searched against three protein databases: UniProt (H. sapiens or P. alecto) , translated Trinity transcriptome and translated Cufflinks transcriptome for (A) PaKiT03 and (B) HEK293T. The number of proteinGroups identified and shared between the databases is given. [file 13059_2014_532_MOESM9_ESM.pdf]
