# Supplementary material for: Proteomics informed by transcriptomics reveals Hendra virus sensitizes bat cells to TRAIL-mediated apoptosis
Source: Genome Biol. 2014 Nov 15;15(11):532. doi: 10.1186/s13059-014-0532-x (PMC4269970; doi:10.1186/s13059-014-0532-x)
Supplement: Additional file 12 — Primer sequences used for this study. [file 13059_2014_532_MOESM12_ESM.docx]

| **Bat (*P. alecto*)** | **Name** | **Primer 3’🡪 5’** |
| --- | --- | --- |
|  | GAPDH-F | ATACTTCTCATGGTTCACAC |
|  | GAPDH-R | TCATTGACCTCAACTACATG |
|  | TNFAIP3-F | CACCCTCAAGGAGACGGATA |
|  | TNFAIP3-R | TGTGGACGCCATTTTGATAA |
|  | TNFRSF11B-F | CCCTGCAGAAAACACACAAA |
|  | TNFRSF11B-R | CTGAACAATGCCTCCTCACA |
|  | TNFRSF10B-F | CAGTGCAAACCTGGCACTTA |
|  | TNFRSF10B-R | TGTGAATTGCCTGATTCTCG |
|  | CD40-F | TTCTCCAACGTGTCATCTGC |
|  | CD40-R | CCATAATGATGGGGATCAGC |
|  | CASP13-F | CACTCGTCTGGCTCTCATCA |
|  | CASP13-R | ATTTCCGTGGCTGTGAGTTT |
|  | TRAIL-F | TGACCTGTGCTCTGATCCTG |
|  | TRAIL-R | CCCAAGAGCTGTCATCTTCC |
| **Human** | GAPDH-F | TGCACCACCAACTGCTTAGC |
|  | GAPDH-R | GGCATGGACTGTGGTCATGAG |
|  | IFNB1-F | GTCAGAGTGGAAATCCTAAG |
|  | IFNB1-R | ACAGCATCTGCTGGTTGAAG |
|  | IFIT2-F | CGTGGGAACCTGGTGACTAA |
|  | IFIT2-R | TCGTTCCAAGCATACCGTGA |
|  | IFIT3-F | GGGCAGACTCTCAGATGCTC |
|  | IFIT3-R | ACCTTCGCCCTTTCATTTCT |

Additional file 12. Primer sequences used in this study.
